# The complete chloroplast genome sequence of a traditional Chinese medicine: *Achyranthes bidentata* (Amaranthaceae)

**DOI:** 10.1080/23802359.2019.1698362

**Published:** 2019-12-11

**Authors:** Zheng-Hui Li, Xiao-Hua Li, Li-Zhen Ling, Hong-Lian Ai, Shu-Dong Zhang

**Affiliations:** a School of Pharmaceutical Sciences, South-Central University for Nationalities, Wuhan, Hubei, China;; b School of Biological Sciences and Technology, Liupanshui Normal University, Liupanshui, Guizhou, China

**Keywords:** *Achyranthes bidentata*, chloroplast genome, medicinal plant, phylogenetic analysis

## Abstract

*Achyranthes bidentata* (Amarathaceae) has been commonly used as a traditional Chinese medicine in the treatment of osteoporosis and bone nonunion. Here, the complete chloroplast genome of *A. bidentata* was assembled and characterized. The cp genome is 151,451 bp in length, composed of a pair of 25,150 bp inverted repeat (IR) regions separated by a large single-copy (LSC) region of 83,899 bp and a small single-copy (SSC) region of 17,252 bp. The whole cp genome of *A*. *bidentata* contains 130 genes(85 protein-coding genes, 37 tRNAs and eight rRNAs) and the overall GC content is 36.5%. Phylogenetic analysis based on the cp genome data showed that *A*. *bidentata* was close to *Cyathula capitata*.

*Achyranthes bidentata* Blume (Amarathaceae), has been used for thousands of years as a blood-activating and stasis-resolving medicine for the treatment of osteoporosis in China and India (He et al. 2017). Moreover, its major component, *A. bidentata* alcohol, has anti-asthmatic, anti-inflammatory, anti-pyretic, anti-rheumatic, and diuretic activities (Hua and Zhang 2019), and *A. bidentata* polypeptides (ABPP) possess neuroprotective activity (Shen et al. 2008; Peng et al. 2018). In this study, we aim to establish and characterize the complete chloroplast (cp) genome of *A. bidentata*, and assess its phylogenetic position within Amaranthaceae.

Fresh and clean leaves of *A. bidentata* were sampled from Longping town of Jianshi county, Hubei, China (N30°48′24″, E110°1′47″, 1,750 m). The voucher specimen (HSN12316) was deposited in the herbarium of South-Central University for Nationalities (HSN). The total genomic DNA was extracted and used for sequencing on Illumina HiSeq 4000 Platform at the Beijing Novogene Bioinformatics Technology Co., Ltd. (Nanjing, China). About 2 GB raw data were used to *de novo* assemble the complete cp genome using SPAdes (Bankevich et al. 2012). The complete genome sequence was annotated using PGA (Qu et al. 2019) with manual adjustments. The sequence of cp genome was deposited in GenBank (accession numbers MN652923).

The circular cp genome of *A. bidentata* is 151,451 bp in size, and exhibits a typical quadripartite structure found in most land plants which is made up of a large single-copy region (LSC) of 83,899 bp, a small single-copy region (SSC) of 17,252 bp, isolated by a pair of identical inverted repeat (IR) regions of 25,150 bp. The total GC content of the whole sequence is 36.5%. The complete cp genome encodes 130 genes, including 85 protein-coding genes, 37 tRNA genes, and eight rRNA genes. Most of the genes occurred in a single copy, while four rRNA genes (i.e. *4.5S*, *5S*, *16S*, and *23S rRNA*), seven tRNA genes (i.e. *trnA-UGC*, *trnI-CAU*, *trnI-GAU*, *trnL-CAA*, *trnN-GUU*, *trnR-ACG*, and *trnV-GAC*), and six protein-coding genes (i.e. *ndhB*, *rpl2*, *rpl23*, *rps7*, *rps12*, and *ycf2*) occurred in double. Among the 113 unique genes, 14 had one intron, and three had two introns (*clpP*, *rps12*, and *ycf3*).

The phylogenetic position of *A. bidentata* was analyzed based on the complete cp genomes of this species and other seventeen species belonging to Achatocarpaceae, Amaranthaceae and Caryophyllaceae. The sequences were aligned with MAFFT (Katoh and Standley 2013). The maximum-likelihood (ML) and Bayesian inference (BI) phylogenetic trees were reconstructed using RAxML (Stamatakis 2014) and MrBayes (Ronquist et al. 2012). The ML and BI analyses generated the same tree topology (Figure 1). As shown in the phylogenetic tree (Figure 1), *A. bidentata* was closely related to *Cyathula capitata* with 100% bootstrap and 1.0 posterior probability support, respectively. Our findings will provide a foundation for further investigation of cp genome evolution and phylogenetic studies of *Achyranthes*.

**Figure 1.**
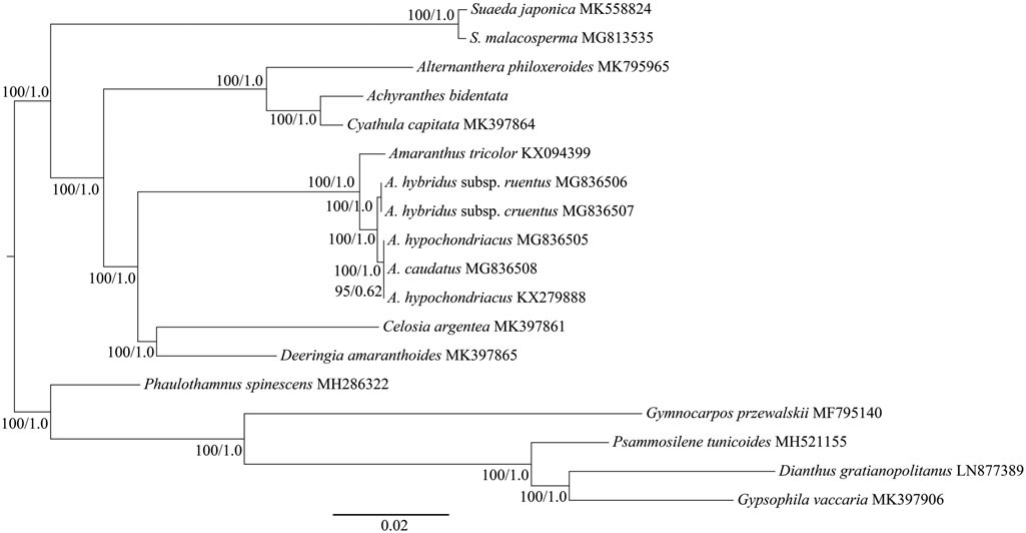
The maximum-likelihood (ML) tree of Amaranthaceae inferred from the complete chloroplast genome sequences. Numbers at nodes correspond to ML bootstrap percentages (1000 replicates) and Bayesian inference (BI) posterior probabilities.
